# Supplementary material for: Cocrystals by Design: A Rational Coformer Selection Approach for Tackling the API Problems
Source: Pharmaceutics. 2023 Apr 6;15(4):1161. doi: 10.3390/pharmaceutics15041161 (PMC10140925; doi:10.3390/pharmaceutics15041161)
Supplement: Supplementary file 1 [file pharmaceutics-15-01161-s001.zip › pharmaceutics-2288495-supplementary.pdf]

Cocrystals by Design: A Rational Coformer Selection Approach for Tackling the API Problems

Maan Singh [1,†], Harsh Barua [2,†], V.G.S.S. Jyothi [3], Madhukiran R. Dhondale [1], Amritha G. Nambiar [1], Ashish K. Agrawal [1], Pradeep Kumar [4], Nalini R. Shastri [5,*] and Dinesh Kumar [1,*]

[1] Pharmaceutical Solid State Research Laboratory, Department of Pharmaceutical Engineering and Technology, Indian Institute of Technology (Banaras Hindu University), Varanasi 221005, India
[2] Solid State Pharmaceutical Cluster (SSPC), Science Foundation Ireland Research Centre for Pharmaceuticals, Bernal Institute, Department of Chemical Sciences, University of Limerick, V94T9PX Limerick, Ireland
[3] Department of Pharmaceutics, National Institute of Pharmaceutical Education and Research, Hyderabad 500037, India
[4] Wits Advanced Drug Delivery Platform Research Unit, Department of Pharmacy and Pharmacology, School of Therapeutic Sciences, Faculty of Health Sciences, University of the Witwatersrand, Johannesburg 2193, South Africa
[5] Consultant, Solid State Pharmaceutical Research, Hyderabad 500037, India
* Correspondence: nalini.niperhyd@gmail.com (N.R.S.); dinesh.phe@itbhu.ac.in (D.K.)
† These authors contributed equally to this work.

Table S1: Reported coformers in literature used in the formation of cocrystals

Drug	Coformer/API Name	Cocrystals	API:Coformer Ratio	Reference
Diclofenac (DIC)	4,4'-Bipyridine (4,4 BPY)	DIC-4,4 BPY Cocrystal	2:1	[1]
5-Fluorouracil (5-FU)	Urea (UA)	5-FU-UA Cocrystal	1:1	[2]
	Thiourea (TUA)	5-FU-TUA Cocrystal	1:1	
	Pyrazinamide (PZA)	5-FU-PZA Cocrystal	2:1	
Carbamazepine (CMP)	Saccharin (SAC)	CMP-SAC Cocrystal	1:1	[3]
Progesterone (PROG)	4-Hydroxybenzoic acid (4-HBA)	PROG-4-HBA Cocrystal	1:1, 1:2, 2:1	[4]
Isoniazid (INH)	2,5-Dihydroxybenzoic acid (2,5-DHBA)	INH-2,5-DHBA Cocrystal	1:1	[5]
	2,4-Dihydroxycinnamic acid (2,4-DHCA)	INH-2,4-DHCA Cocrystal	1:1	
Imidazopyridazine (IMP)	Succinic Acid (SA)	IMP-SA Cocrystal	2:1, 1:1	[6]
Itraconazole (ITZ)	Succinic Acid (SA)	ITZ-SA Cocrystals	2:1	[7]
	Fumaric acid (FA)	ITZ-FA Cocrystals	2:1	
Carbamazepine (CMP)	Nicotinamide (NTA)	CMP-NTA Cocrystals	1:1	
Itraconazole (ITZ)	Tartaric Acid (TA)	ITZ-TA Cocrystals	2:1	[8]

	Malic acid (MA)	ITZ-MA Cocrystals	2:1	
Myricetin (MYR)	Isonicotinamide (INA)	MYR-INA Cocrystals	1:2	[9]
	Caffeine (CAF)	MYR-CAF Cocrystals	1:1	
Adefovir dipivoxil (AD)	Saccharin (SAC)	AD-SAC Cocrystals	1:1	[10]
	Nicotinamide (NTA)	AD-NTA Cocrystals	1:1	
Theophylline (THP)	Citric acid (CTA)	THP-CTA Cocrystals	1:1	[11]
Carbamazepine (CBZ)	4 Amino benzoic acid (4ABA)	CBZ-4ABA Cocrystals	2:1	
Indomethacin (IMC)	Saccharin (SAC)	IMC-SAC Cocrystal	1:1	[12]
Anhydrous theophylline (TP)	Citric acid (CA)	TP-CA Cocrystals	1:1	
Carbamazepine (CBZ)	Saccharin (SAC)	CBZ–SAC Cocrystals	1:1	[13]
	Cinnamic acid	CBZ–CIN Cocrystals	1:1	
Carbamazepine (CBZ)	Nicotinamide (NTA)	CBZ-NTA Cocrystals	1:1	[14]
Meloxicam	Aspirin	MLX-APN Cocrystals	1:1	[15]
Efavirenz (EFV)	DL-alanine (ALA)	EFV-ALA Cocrystals	1:1	[16]
	Oxalic acid (OX)	EFV-OX Cocrystals	1:1	
	Maleic acid (MAL)	EFV-MAL Cocrystals	1:1	
	Nicotinamide (NIC)	EFV-NIC Cocrystals	1:1	
Hydrochlorothiazide	Nicotinamide (NCT)	HTZ-NCT Cocrystals	1:1	[17]
Myricetin (MYR)	Nicotinamide (NCT)	MYR-NCT Cocrystals	1:2	[18]
	Proline (PRO)	MYR-PRO Cocrystals	1:2	
Quercetin (QUE)	Caffeine (CAF)	QUE-CAF Cocrystals	1:1	
	Nicotinamide (NCT)	QUE-NCT Cocrystals	1:1	
	Theobromine (TBR)	QUE-TBR Cocrystals	1:1	
Curcumin (CUR)	Ascorbic acid (AA)	CUR-AA Cocrystals	-	[19]
Carvedilol (CAR)	Tartaric Acid (TA)	CAR-TA Cocrystals	1:1	[20]
Efavirenz (EFV)	Tartaric Acid (TA)	EFV-TA Cocrystals	1:1	[21]
	Adipic acid (ADP)	EFV-ADP Cocrystals	1:1	

Brexpiprazole (BPZ)	Fumaric acid (FUM)	BPZ-FUM Cocrystals	1:1	[22]
	Malonic acid (MAL)	BPZ-MAL Cocrystals	1:1	
	Succinic acid (SUC)	BPZ-SUC Cocrystals	1:1	
	Glutaric acid (GLU)	BPZ-GLU Cocrystals	1:1	
Aripiprazole (ARP)	Succinic acid (SA)	ARP-SA Cocrystals	1:1	[23]
	Nicotinamide (NCT)	ARP-NCT Cocrystals	1:1	
Ketoconazole (KTZ)	Succinic acid (SUC)	KTZ-SA Cocrystals	1:1	[24]
	Fumaric acid (FUM)	KTZ-FUM Cocrystals	1:1	
	Adipic acid (ADP)	KTZ-ADP Cocrystals	1:1	
Abiraterone acetate (ABI)	Succinic acid (SA)	ABI-SA Cocrystals	2:1	[25]
	Glutaric acid (GA)	ABI-GA Cocrystals	1:1	
	4-Hydroxy benzoic acid (4-HBA)	ABI-4-HBA Cocrystals	1:1	
	3,5-Dihydroxy benzoic acid (3,5-DHBA)	ABI-3,5-DHBA Cocrystals	1:1	
Sulfamethazine (SZ)	2-Chloro-4-nitrobenzoic acid (2C4N)	SZ-2C4N Cocrystals	1:1	[26]
	2-Chloro-5-nitrobenzoic acid (2C5N)	SZ-2C5N Cocrystals	1:1	
	Salicylic acid (2HBA)	SZ-2HBA Cocrystals	1:1	
	3-Hydroxybenzoic acid (3HBA)	SZ-3HBA Cocrystals	1:1	
	4-Bromobenzoic acid (4BRBA)	SZ-4BRBA Cocrystals	1:1	
	Benzoic acid (BA)	SZ-BA Cocrystals	1:1	
	Cinnamic acid (CA)	SZ-CA Cocrystals	1:1	
	Toluic acid (TA)	SZ-TA Cocrystals	1:1	
Acyclovir (ACV)	Succinic acid (SA)	ACV-SA Cocrystals	1:1	[27]
Fluoxetine (FHC)	Succinic acid (SA)	FHC-SA Cocrystals	1:1	
Entacapone (ETP)	Acetamide (ACT)	ETP-ACT Cocrystals	1:1	[28]
	Nicotinamide (NAM)	ETP-NAM Cocrystals	1:1	
	Isonicotinamide (INAM)	ETP-INAM Cocrystals	1:1	

	Pyrazinamide (PYZ)	ETP-PYZ Cocrystals	1:1	
	Isoniazid (INZ)	ETP-INZ Cocrystals	1:1	
Pyrazinamide (PZA)	4-Hydroxy benzoic acid (4-HBA)	PZA-4-HBA Cocrystals	1:1	[29]
	p-Coumaric acid (PCA)	PZA-PCA Cocrystals	-	
	Ferulic acid (FRA)	PZA-FRA Cocrystals	1:1	
	Sinapic acid (SPA)	PZA-SPA Cocrystals	1:1	
3,5-Dinitrobenzoic acid (DNBA)	3-cyanopyridine (3-CNP)	DNBA-3-CNP Cocrystals	1:1	[30]
	Flufenamic acid (FA)	DNBA-FA Cocrystals	1:1	
	Theophylline (THP)	DNBA-THP Cocrystals	1:1	
	Thiourea (TU)	DNBA-TU Cocrystals	1:1	
Theophylline (THP)	Glutaric acid (GA)	THP-GA Cocrystals	1:1	[31]
	Isonicotinamide (INAM)	THP-INAM Cocrystals	1:1	
	Benzamide (BZ)	THP-BZ Cocrystals	1:1	
Oxyresveratrol (OXY)	Nicotinamide (NCT)	OXY-NCT Cocrystals	1:1	[32]
	Proline (PRO)	OXY-PRO Cocrystals	1:1	

1. Goswami, P.K.; Kumar, V.; Ramanan, A. Multicomponent solids of diclofenac with pyridine based coformers. *Journal of Molecular Structure* **2020**, *1210*, doi:10.1016/j.molstruc.2020.128066.
2. Cuadra, I.A.; Cabañas, A.; Cheda, J.A.R.; Türk, M.; Pando, C. Cocrystallization of the anticancer drug 5-fluorouracil and coformers urea, thiourea or pyrazinamide using supercritical CO2 as an antisolvent (SAS) and as a solvent (CSS). *The Journal of Supercritical Fluids* **2020**, *160*, doi:10.1016/j.supflu.2020.104813.
3. Budiman, A.; Higashi, K.; Ueda, K.; Moribe, K. Effect of drug-coformer interactions on drug dissolution from a coamorphous in mesoporous silica. *Int J Pharm* **2021**, *600*, 120492, doi:10.1016/j.ijpharm.2021.120492.
4. Samipillai, M.; Rohani, S. The role of higher coformer stoichiometry ratio in pharmaceutical cocrystals for improving their solid-state properties: The cocrystals of progesterone and 4-hydroxybenzoic acid. *Journal of Crystal Growth* **2019**, *507*, 270-282, doi:10.1016/j.jcrysgro.2018.10.050.
5. Mashhadi, S.M.A.; Yunus, U.; Bhatti, M.H. Structural characterization and in-situ synthesis of quaternary ionic-cocrystal of isoniazid from un-ionized coformers. *Journal of Molecular Structure* **2021**, *1233*, doi:10.1016/j.molstruc.2021.130015.
6. Noonan, T.J.; Chibale, K.; Bourne, S.A.; Caira, M.R. A preformulation co-crystal screening case study: Polymorphic co-crystals of an imidazopyridazine antimalarial drug lead with the coformer succinic acid. *Journal of Molecular Structure* **2020**, *1204*, doi:10.1016/j.molstruc.2019.127561.

7. Patil, S.; Ujalambkar, V.; Mahadik, A. Electrospray technology as a probe for cocrystal synthesis: Influence of solvent and coformer structure. *Journal of Drug Delivery Science and Technology* **2017**, *39*, 217-222, doi:10.1016/j.jddst.2017.04.001.
8. Abramov, Y.A.; Loschen, C.; Klamt, A. Rational coformer or solvent selection for pharmaceutical cocrystallization or desolvation. *J Pharm Sci* **2012**, *101*, 3687-3697, doi:10.1002/jps.23227.
9. Ren, S.; Liu, M.; Hong, C.; Li, G.; Sun, J.; Wang, J.; Zhang, L.; Xie, Y. The effects of pH, surfactant, ion concentration, coformer, and molecular arrangement on the solubility behavior of myricetin cocrystals. *Acta Pharmaceutica Sinica B* **2019**, *9*, 59-73, doi:10.1016/j.apsb.2018.09.008.
10. Gao, Y.; Gao, J.; Liu, Z.; Kan, H.; Zu, H.; Sun, W.; Zhang, J.; Qian, S. Coformer selection based on degradation pathway of drugs: A case study of adefovir dipivoxil–saccharin and adefovir dipivoxil–nicotinamide cocrystals. *International Journal of Pharmaceutics* **2012**, *438*, 327-335, doi:10.1016/j.ijpharm.2012.09.027.
11. Jayasankar, A.; Roy, L.; Rodriguez-Hornedo, N. Transformation pathways of cocrystal hydrates when coformer modulates water activity. *J Pharm Sci* **2010**, *99*, 3977-3985, doi:10.1002/jps.22245.
12. Lin, H.-L.; Zhang, G.-C.; Hsu, P.-C.; Lin, S.-Y. A portable fiber-optic Raman analyzer for fast real-time screening and identifying cocrystal formation of drug-coformer via grinding process. *Microchemical Journal* **2013**, *110*, 15-20, doi:10.1016/j.microc.2013.01.004.
13. Qiu, S.; Li, M. Effects of coformers on phase transformation and release profiles of carbamazepine cocrystals in hydroxypropyl methylcellulose based matrix tablets. *Int J Pharm* **2015**, *479*, 118-128, doi:10.1016/j.ijpharm.2014.12.049.
14. Tomaszewska, I.; Karki, S.; Shur, J.; Price, R.; Fotaki, N. Pharmaceutical characterisation and evaluation of cocrystals: Importance of in vitro dissolution conditions and type of coformer. *International Journal of Pharmaceutics* **2013**, *453*, 380-388, doi:10.1016/j.ijpharm.2013.05.048.
15. Cheney, M.L.; Weyna, D.R.; Shan, N.; Hanna, M.; Wojtas, L.; Zaworotko, M.J. Coformer selection in pharmaceutical cocrystal development: a case study of a meloxicam aspirin cocrystal that exhibits enhanced solubility and pharmacokinetics. *J Pharm Sci* **2011**, *100*, 2172-2181, doi:10.1002/jps.22434.
16. Gowda, B.H.J.; Ahmed, M.G.; Shankar, S.J.; Paul, K.; Chandan, R.S.; Sanjana, A.; Narayana, S.; Nasrine, A.; Noushida, N.; Thriveni, M. Preparation and characterization of efavirenz cocrystals: An endeavor to improve the physicochemical parameters. *Materials Today: Proceedings* **2022**, *57*, 878-886, doi:10.1016/j.matpr.2022.02.543.
17. Queiroz, A.L.P.; Rodrigues, M.; Zeglinski, J.; Crean, A.M.; Sarraguça, M.C.; Vucen, S. Determination of co-crystal phase purity by mid infrared spectroscopy and multiple curve resolution. *International Journal of Pharmaceutics* **2021**, *595*, doi:10.1016/j.ijpharm.2021.120246.
18. Dias, J.L.; Lanza, M.; Ferreira, S.R.S. Cocrystallization: A tool to modulate physicochemical and biological properties of food-relevant polyphenols. *Trends in Food Science & Technology* **2021**, *110*, 13-27, doi:10.1016/j.tifs.2021.01.035.
19. Pantwalawalkar, J.; More, H.; Bhange, D.; Patil, U.; Jadhav, N. Novel curcumin ascorbic acid cocrystal for improved solubility. *Journal of Drug Delivery Science and Technology* **2021**, *61*, doi:10.1016/j.jddst.2020.102233.
20. Mohammady, M.; Hadidi, M.; Iman Ghetmiri, S.; Yousefi, G. Design of ultra-fine carvedilol nanococrystals: Development of a safe and stable injectable formulation. *European Journal of Pharmaceutics and Biopharmaceutics* **2021**, *168*, 139-151, doi:10.1016/j.ejpb.2021.08.015.
21. Gowda, B.H.J.; Nechipadappu, S.K.; Shankar, S.J.; Chavali, M.; Paul, K.; Ahmed, M.G.; Sanjana, A.; Shanthala, H.K. Pharmaceutical cocrystals of Efavirenz: Towards the improvement of solubility, dissolution rate and stability. *Materials Today: Proceedings* **2022**, *51*, 394-402, doi:10.1016/j.matpr.2021.05.535.
22. Rama, V.; Vidavulur, S.; Tadikonda, P.V.; Rajana, N.; Mittapalli, S. Novel cocrystals of brexpiprazole with improved solubility. *Journal of Crystal Growth* **2020**, *551*, doi:10.1016/j.jcrysgro.2020.125910.

23. Butreddy, A.; Almutairi, M.; Komanduri, N.; Bandari, S.; Zhang, F.; Repka, M.A. Multicomponent crystalline solid forms of aripiprazole produced via hot melt extrusion techniques: An exploratory study. *Journal of Drug Delivery Science and Technology* **2021**, *63*, doi:10.1016/j.jddst.2021.102529.
24. Cao, F.; Rodriguez-Hornedo, N.; Amidon, G.E. Mechanistic Analysis of Cocrystal Dissolution, Surface pH, and Dissolution Advantage as a Guide for Rational Selection. *Journal of Pharmaceutical Sciences* **2019**, *108*, 243-251, doi:10.1016/j.xphs.2018.09.028.
25. Chennuru, R.; Devarapalli, R.; Rengaraj, P.; Srinivas, P.L.; Dey, S.; Reddy, C.M. Improving Solubility of Poorly Soluble Abiraterone Acetate by Cocrystal Design Aided by In Silico Screening. *Crystal Growth & Design* **2020**, *20*, 5018-5030, doi:10.1021/acs.cgd.0c00153.
26. Scheepers, M.C.; Lemmerer, A. Synthesis and Characterization of a Series of Sulfamethazine Multicomponent Crystals with Various Benzoic Acids. *Crystal Growth & Design* **2019**, *20*, 813-823, doi:10.1021/acs.cgd.9b01209.
27. Rahman, F.; Winantari, A.N.; Setyawan, D.; Siswandono. Comparison Study of Grinding and Slurry Method on Physicochemical Characteristic of Acyclovir – Succinic Acid Cocrystal. *Asian Journal of Pharmaceutical and Clinical Research* **2017**, *10*, doi:10.22159/ajpcr.2017.v10i3.15925.
28. Bommaka, M.K.; Mannava, M.K.C.; Suresh, K.; Gunnam, A.; Nangia, A. Entacapone: Improving Aqueous Solubility, Diffusion Permeability, and Cocrystal Stability with Theophylline. *Crystal Growth & Design* **2018**, *18*, 6061-6069, doi:10.1021/acs.cgd.8b00921.
29. Rajbongshi, T.; Sarmah, K.K.; Sarkar, A.; Ganduri, R.; Cherukuvada, S.; Thakur, T.S.; Thakuria, R. Preparation of Pyrazinamide Eutectics versus Cocrystals Based on Supramolecular Synthon Variations. *Crystal Growth & Design* **2018**, *18*, 6640-6651, doi:10.1021/acs.cgd.8b00878.
30. Scheepers, M.C.; Lemmerer, A. Exploring the Crystal Structure Landscape of 3,5-Dinitrobenzoic Acid through Various Multicomponent Molecular Complexes. *Crystal Growth & Design* **2020**, *21*, 344-356, doi:10.1021/acs.cgd.0c01217.
31. Koranne, S.; Krzyzaniak, J.F.; Luthra, S.; Arora, K.K.; Suryanarayanan, R. Role of Coformer and Excipient Properties on the Solid-State Stability of Theophylline Cocrystals. *Crystal Growth & Design* **2019**, *19*, 868-875, doi:10.1021/acs.cgd.8b01430.
32. Ouiyangkul, P.; Tantishaiyakul, V.; Hirun, N. Exploring potential coformers for oxyresveratrol using principal component analysis. *International Journal of Pharmaceutics* **2020**, *587*, doi:10.1016/j.ijpharm.2020.119630.
